# [4-Carboxy­imidazole-5-carboxyl­ato(2–)-κ^2^ *N* ^1^,*O* ^5^](5,5,7,12,12,14-hexa­methyl-1,4,8,11-tetra­azacyclo­tetra­decane-κ^4^ *N*,*N*′,*N*′′,*N*′′′)nickel(II) monohydrate

**DOI:** 10.1107/S1600536809046145

**Published:** 2009-11-11

**Authors:** Guang-Chuan Ou, Qiang Zhou, Seik Weng Ng

**Affiliations:** a Department of Biology and Chemistry, Hunan University of Science and Engineering, Yongzhou, Hunan 425100, People’s Republic of China; b Department of Chemistry, University of Malaya, 50603 Kuala Lumpur, Malaysia

## Abstract

The 4-carboxy­imidazole-5-carboxyl­ate(2−) dianion in the title compound, [Ni(C_5_H_2_N_2_O_4_)(C_16_H_36_N_4_)]·H_2_O, *N*,*O*′-chelates to the Ni^II^ atom, which shows an octa­hedral coordination. The macrocycle folds itself around the metal atom and binds to it through four secondary nitrogen atoms; adjacent molecules are linked by N—H⋯O hydrogen bonds into a linear chain. The water molecule is disordered over two positions.

## Related literature

For the crystal structures of other nickel salts of the macrocycle, see: Ou *et al.* (2009*a* ▶,*b* ▶).
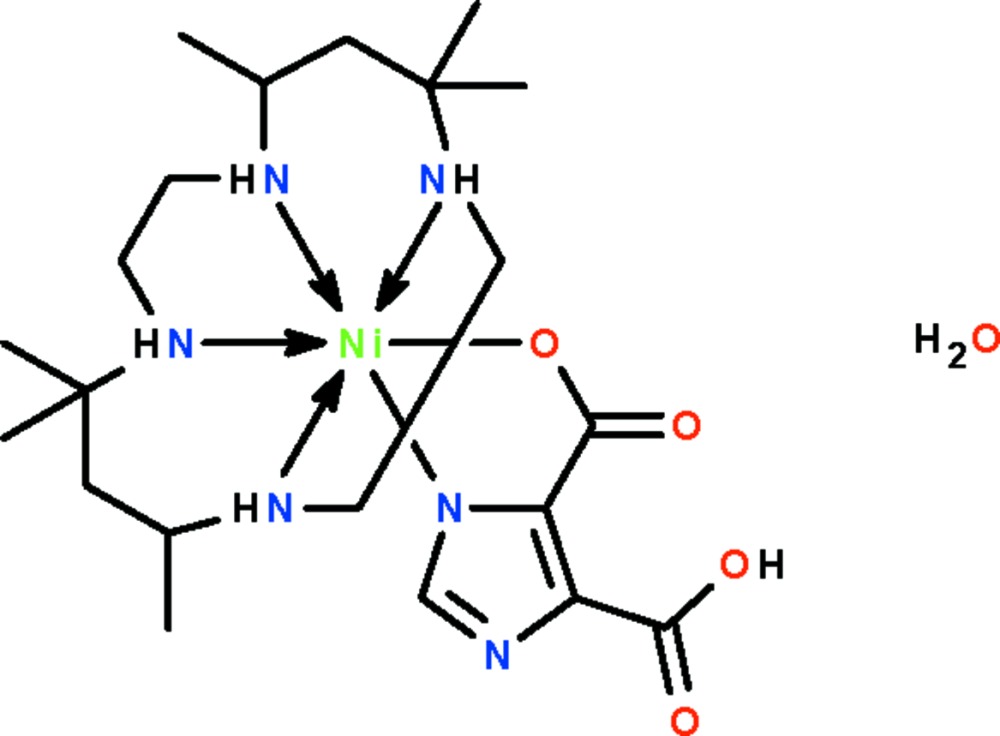


## Experimental

### 

#### Crystal data

[Ni(C_5_H_2_N_2_O_4_)(C_16_H_36_N_4_)]·H_2_O
*M* *_r_* = 515.30
Monoclinic, 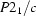

*a* = 10.4070 (8) Å
*b* = 12.7806 (10) Å
*c* = 19.0947 (14) Å
β = 103.675 (1)°
*V* = 2467.7 (3) Å^3^
*Z* = 4
Mo *K*α radiation
μ = 0.83 mm^−1^
*T* = 295 K
0.42 × 0.36 × 0.12 mm

#### Data collection

Bruker SMART area-detector diffractometer
Absorption correction: multi-scan (*SADABS*; Sheldrick, 1996 ▶) *T*_min_ = 0.767, *T*_max_ = 0.905
10408 measured reflections
4308 independent reflections
2809 reflections with *I* > 2σ(*I*)
*R*_int_ = 0.046

#### Refinement

*R*[*F* ^2^ > 2σ(*F* ^2^)] = 0.049
*wR*(*F* ^2^) = 0.163
*S* = 1.04
4308 reflections
309 parameters
12 restraints
H-atom parameters constrained
Δρ_max_ = 0.70 e Å^−3^
Δρ_min_ = −0.36 e Å^−3^

### 

Data collection: *SMART* (Bruker, 1999 ▶); cell refinement: *SAINT-Plus* (Bruker, 1999 ▶); data reduction: *SAINT-Plus*; program(s) used to solve structure: *SHELXS97* (Sheldrick, 2008 ▶); program(s) used to refine structure: *SHELXL97* (Sheldrick, 2008 ▶); molecular graphics: *X-SEED* (Barbour, 2001 ▶); software used to prepare material for publication: *publCIF* (Westrip, 2009 ▶).

## Supplementary Material

Crystal structure: contains datablocks I, global. DOI: 10.1107/S1600536809046145/xu2666sup1.cif

Structure factors: contains datablocks I. DOI: 10.1107/S1600536809046145/xu2666Isup2.hkl

Additional supplementary materials:  crystallographic information; 3D view; checkCIF report

## Figures and Tables

**Fig. 1.**
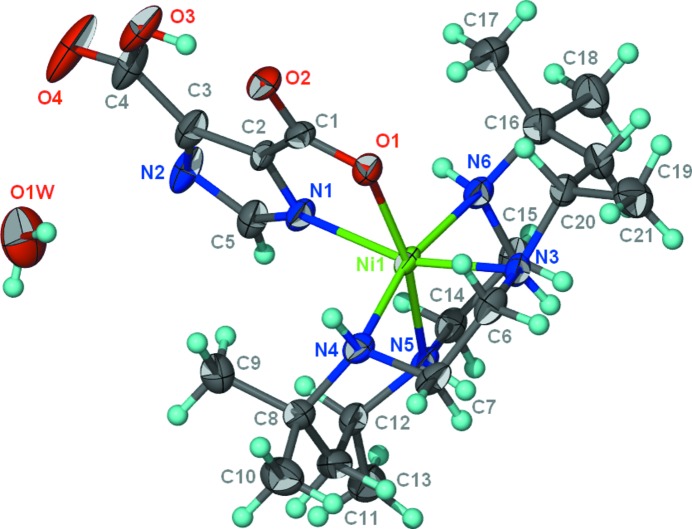
Thermal ellipsoid plot (Barbour, 2001) of Ni(C16H36N4)(C5H2N2O4).H2O showing displacement ellipsoids of the 50% probability level. H-atoms are drawn as spheres of arbitrary radii.

## supplementary crystallographic information

### Experimental

Imidazole-4,5-dicarboxylic acid (0.156 g, 1 mmol) and sodium hydroxide (0.080 g, 2 mmol) were dissolved in water (5 ml). To this solution was added (5,5,7,12,12,14 -hexamethyl-1,4,8,11-tetraazacyclotetradecane)nickel perchlorate (0.54 g, 1 mmol) dissolved in acetonitrile (10 ml). The solution was left to stand at room temperature; blue crystals formed after several weeks.

### Refinement

Carbon-, nitrogen- and oxygen-bound H-atoms were positioned geometrically and refined using the riding model, and with C—H = 0.93 to 0.98 Å, N–H 0.86 Å, O–H 0.84 Å and *U*(H) set to 1.2–1.5 *U*_eq_(C,*N*,*O*).

The water molecule does not form a hydrogen bond to other acceptor atoms. It is disordered over two positions in a 60:40 ratio. The anisotropic temperature factors were restrained to be nearly isotropic. The H-atoms were positioned so that there are no short H···H contacts.

### Crystal data

**Table:**

[Ni(C_5_H_2_N_2_O_4_)(C_16_H_36_N_4_)]·H_2_O	*F*(000) = 1104
*M**_r_* = 515.30	*D*_x_ = 1.387 Mg m^−^^3^
Monoclinic, *P*2_1_/*c*	Mo *K*α radiation, λ = 0.71073 Å
Hall symbol: -P 2ybc	Cell parameters from 2768 reflections
*a* = 10.4070 (8) Å	θ = 2.6–24.5°
*b* = 12.7806 (10) Å	µ = 0.83 mm^−^^1^
*c* = 19.0947 (14) Å	*T* = 295 K
β = 103.675 (1)°	Block, blue
*V* = 2467.7 (3) Å^3^	0.42 × 0.36 × 0.12 mm
*Z* = 4	

### Data collection

**Table:**

Bruker SMART area-detector diffractometer	4308 independent reflections
Radiation source: fine-focus sealed tube	2809 reflections with *I* > 2σ(*I*)
graphite	*R*_int_ = 0.046
φ and ω scans	θ_max_ = 25.0°, θ_min_ = 1.9°
Absorption correction: multi-scan (*SADABS*; Sheldrick, 1996)	*h* = −12→12
*T*_min_ = 0.767, *T*_max_ = 0.905	*k* = −15→6
10408 measured reflections	*l* = −22→22

### Refinement

**Table:**

Refinement on *F*^2^	Primary atom site location: structure-invariant direct methods
Least-squares matrix: full	Secondary atom site location: difference Fourier map
*R*[*F*^2^ > 2σ(*F*^2^)] = 0.049	Hydrogen site location: inferred from neighbouring sites
*wR*(*F*^2^) = 0.163	H-atom parameters constrained
*S* = 1.04	*w* = 1/[σ^2^(*F*_o_^2^) + (0.0959*P*)^2^ + 0.1761*P*] where *P* = (*F*_o_^2^ + 2*F*_c_^2^)/3
4308 reflections	(Δ/σ)_max_ = 0.001
309 parameters	Δρ_max_ = 0.70 e Å^−^^3^
12 restraints	Δρ_min_ = −0.36 e Å^−^^3^

### Fractional atomic coordinates and isotropic or equivalent isotropic displacement parameters (Å^2^)

**Table:**

	*x*	*y*	*z*	*U*_iso_*/*U*_eq_	Occ. (<1)
Ni1	0.30812 (5)	0.45585 (4)	0.28750 (3)	0.0245 (2)	
O1	0.4614 (3)	0.5510 (3)	0.34989 (16)	0.0336 (8)	
O2	0.6725 (3)	0.5949 (3)	0.36493 (17)	0.0376 (8)	
O3	0.8653 (3)	0.5250 (3)	0.3258 (2)	0.0538 (11)	
H3O	0.7997	0.5424	0.3417	0.081*	
O4	0.9127 (4)	0.4070 (3)	0.2526 (3)	0.0829 (16)	
O1W	0.7320 (8)	0.3543 (8)	0.0802 (4)	0.073 (3)	0.601 (16)
H1W1	0.7148	0.4042	0.1052	0.110*	0.601 (16)
H1W2	0.6820	0.3546	0.0387	0.110*	0.601 (16)
O1W'	0.7294 (15)	0.4398 (15)	0.0802 (7)	0.091 (6)	0.399 (16)
H1W3	0.7297	0.4873	0.1107	0.136*	0.399 (16)
H1W4	0.6785	0.4513	0.0397	0.136*	0.399 (16)
N1	0.4737 (3)	0.3997 (3)	0.2535 (2)	0.0305 (9)	
N2	0.6458 (4)	0.3371 (3)	0.2133 (2)	0.0426 (11)	
N3	0.1845 (3)	0.5172 (3)	0.35147 (19)	0.0278 (9)	
H3N	0.1082	0.4879	0.3381	0.033*	
N4	0.2535 (4)	0.5960 (3)	0.22445 (19)	0.0298 (9)	
H4N	0.3176	0.6389	0.2400	0.036*	
N5	0.1663 (3)	0.3764 (3)	0.20915 (18)	0.0267 (9)	
H5N	0.0897	0.3956	0.2140	0.032*	
N6	0.3160 (3)	0.3119 (3)	0.34868 (19)	0.0303 (9)	
H6N	0.3832	0.2783	0.3413	0.036*	
C1	0.5726 (4)	0.5398 (4)	0.3369 (2)	0.0304 (11)	
C2	0.5852 (4)	0.4563 (3)	0.2869 (2)	0.0261 (10)	
C3	0.6903 (5)	0.4169 (4)	0.2603 (3)	0.0380 (12)	
C4	0.8316 (5)	0.4491 (4)	0.2793 (3)	0.0464 (14)	
C5	0.5175 (4)	0.3311 (4)	0.2119 (3)	0.0360 (12)	
H5	0.4620	0.2821	0.1839	0.043*	
C6	0.1697 (4)	0.6284 (4)	0.3314 (3)	0.0337 (11)	
H6A	0.2505	0.6657	0.3531	0.040*	
H6B	0.0982	0.6587	0.3492	0.040*	
C7	0.1404 (4)	0.6395 (4)	0.2504 (2)	0.0332 (11)	
H7A	0.0600	0.6019	0.2284	0.040*	
H7B	0.1279	0.7127	0.2371	0.040*	
C8	0.2339 (4)	0.5968 (4)	0.1448 (2)	0.0346 (12)	
C9	0.3679 (5)	0.5815 (4)	0.1280 (3)	0.0444 (13)	
H9A	0.4262	0.6369	0.1497	0.067*	
H9B	0.3582	0.5825	0.0767	0.067*	
H9C	0.4043	0.5155	0.1469	0.067*	
C10	0.1777 (5)	0.7000 (4)	0.1105 (3)	0.0493 (14)	
H10A	0.2363	0.7561	0.1303	0.074*	
H10B	0.0925	0.7121	0.1202	0.074*	
H10C	0.1688	0.6966	0.0593	0.074*	
C11	0.1364 (5)	0.5088 (4)	0.1118 (2)	0.0375 (12)	
H11A	0.0524	0.5245	0.1232	0.045*	
H11B	0.1224	0.5134	0.0598	0.045*	
C12	0.1699 (4)	0.3955 (4)	0.1330 (2)	0.0331 (11)	
H12	0.2603	0.3823	0.1284	0.040*	
C13	0.0766 (5)	0.3224 (4)	0.0799 (3)	0.0425 (13)	
H13A	0.0981	0.2510	0.0931	0.064*	
H13B	0.0870	0.3346	0.0320	0.064*	
H13C	−0.0132	0.3362	0.0817	0.064*	
C14	0.1822 (4)	0.2652 (4)	0.2291 (2)	0.0319 (11)	
H14A	0.2589	0.2371	0.2150	0.038*	
H14B	0.1050	0.2264	0.2038	0.038*	
C15	0.1992 (4)	0.2524 (4)	0.3093 (2)	0.0353 (11)	
H15A	0.1207	0.2775	0.3230	0.042*	
H15B	0.2102	0.1789	0.3219	0.042*	
C16	0.3346 (5)	0.3134 (4)	0.4296 (3)	0.0367 (12)	
C17	0.4744 (5)	0.3503 (4)	0.4619 (3)	0.0428 (13)	
H17A	0.4866	0.4188	0.4439	0.064*	
H17B	0.5361	0.3026	0.4488	0.064*	
H17C	0.4892	0.3527	0.5134	0.064*	
C18	0.3178 (5)	0.2034 (4)	0.4592 (3)	0.0491 (14)	
H18A	0.3817	0.1568	0.4471	0.074*	
H18B	0.2303	0.1780	0.4382	0.074*	
H18C	0.3309	0.2067	0.5106	0.074*	
C19	0.2315 (5)	0.3859 (4)	0.4496 (2)	0.0375 (12)	
H19A	0.1447	0.3588	0.4267	0.045*	
H19B	0.2410	0.3808	0.5012	0.045*	
C20	0.2335 (4)	0.5008 (4)	0.4307 (2)	0.0318 (11)	
H20	0.3255	0.5248	0.4445	0.038*	
C21	0.1539 (5)	0.5646 (4)	0.4740 (3)	0.0475 (14)	
H21A	0.1891	0.5531	0.5245	0.071*	
H21B	0.0630	0.5431	0.4610	0.071*	
H21C	0.1598	0.6377	0.4634	0.071*	

### Atomic displacement parameters (Å^2^)

**Table:**

	*U*^11^	*U*^22^	*U*^33^	*U*^12^	*U*^13^	*U*^23^
Ni1	0.0140 (3)	0.0301 (3)	0.0302 (3)	−0.0013 (2)	0.0066 (2)	−0.0022 (3)
O1	0.0243 (17)	0.048 (2)	0.0290 (17)	−0.0063 (15)	0.0068 (13)	−0.0025 (15)
O2	0.0233 (18)	0.045 (2)	0.0416 (19)	−0.0127 (15)	0.0028 (14)	0.0012 (16)
O3	0.0214 (18)	0.044 (2)	0.097 (3)	−0.0017 (17)	0.015 (2)	0.006 (2)
O4	0.031 (2)	0.055 (3)	0.174 (5)	−0.006 (2)	0.047 (3)	−0.025 (3)
O1W	0.091 (5)	0.066 (7)	0.076 (5)	0.014 (4)	0.047 (4)	−0.015 (4)
O1W'	0.117 (9)	0.092 (11)	0.081 (7)	−0.016 (7)	0.058 (6)	0.004 (6)
N1	0.020 (2)	0.036 (2)	0.037 (2)	0.0031 (17)	0.0096 (17)	0.0019 (19)
N2	0.026 (2)	0.035 (2)	0.073 (3)	0.0018 (18)	0.025 (2)	−0.005 (2)
N3	0.0220 (19)	0.033 (2)	0.031 (2)	−0.0021 (16)	0.0121 (16)	−0.0006 (17)
N4	0.022 (2)	0.033 (2)	0.032 (2)	−0.0036 (17)	0.0039 (16)	−0.0001 (18)
N5	0.0155 (18)	0.033 (2)	0.032 (2)	0.0015 (15)	0.0064 (15)	−0.0024 (17)
N6	0.022 (2)	0.034 (2)	0.036 (2)	0.0042 (17)	0.0088 (16)	0.0037 (18)
C1	0.020 (2)	0.037 (3)	0.032 (3)	−0.001 (2)	0.0020 (19)	0.010 (2)
C2	0.016 (2)	0.029 (2)	0.034 (2)	−0.0014 (19)	0.0083 (18)	0.008 (2)
C3	0.025 (3)	0.034 (3)	0.058 (3)	0.004 (2)	0.015 (2)	0.009 (3)
C4	0.026 (3)	0.029 (3)	0.090 (4)	0.000 (2)	0.025 (3)	0.007 (3)
C5	0.029 (3)	0.038 (3)	0.044 (3)	0.000 (2)	0.015 (2)	0.001 (2)
C6	0.025 (2)	0.032 (3)	0.046 (3)	0.003 (2)	0.013 (2)	−0.005 (2)
C7	0.020 (2)	0.030 (3)	0.048 (3)	0.002 (2)	0.005 (2)	0.002 (2)
C8	0.026 (3)	0.044 (3)	0.033 (3)	−0.002 (2)	0.004 (2)	0.008 (2)
C9	0.043 (3)	0.053 (3)	0.040 (3)	−0.006 (3)	0.014 (2)	0.006 (3)
C10	0.052 (3)	0.048 (3)	0.044 (3)	−0.005 (3)	0.004 (3)	0.009 (3)
C11	0.028 (3)	0.053 (3)	0.029 (3)	0.005 (2)	0.003 (2)	0.002 (2)
C12	0.022 (2)	0.047 (3)	0.031 (3)	−0.002 (2)	0.0092 (19)	−0.004 (2)
C13	0.036 (3)	0.053 (3)	0.038 (3)	−0.004 (2)	0.007 (2)	−0.011 (2)
C14	0.026 (2)	0.033 (3)	0.037 (3)	−0.006 (2)	0.009 (2)	−0.006 (2)
C15	0.035 (3)	0.028 (3)	0.047 (3)	−0.007 (2)	0.018 (2)	0.001 (2)
C16	0.034 (3)	0.039 (3)	0.039 (3)	0.003 (2)	0.013 (2)	0.007 (2)
C17	0.031 (3)	0.051 (3)	0.044 (3)	0.002 (2)	0.004 (2)	0.004 (3)
C18	0.054 (3)	0.048 (3)	0.050 (3)	0.004 (3)	0.021 (3)	0.012 (3)
C19	0.037 (3)	0.051 (3)	0.027 (3)	−0.003 (2)	0.012 (2)	0.001 (2)
C20	0.024 (2)	0.048 (3)	0.025 (2)	−0.007 (2)	0.0079 (19)	−0.007 (2)
C21	0.052 (3)	0.058 (4)	0.035 (3)	0.010 (3)	0.014 (2)	−0.005 (3)

### Geometric parameters (Å, °)

**Table:**

Ni1—N5	2.098 (3)	C8—C9	1.516 (7)
Ni1—N1	2.106 (4)	C8—C10	1.527 (7)
Ni1—N3	2.123 (3)	C8—C11	1.545 (7)
Ni1—O1	2.132 (3)	C9—H9A	0.9600
Ni1—N4	2.158 (4)	C9—H9B	0.9600
Ni1—N6	2.171 (4)	C9—H9C	0.9600
O1—C1	1.247 (5)	C10—H10A	0.9600
O2—C1	1.265 (5)	C10—H10B	0.9600
O3—C4	1.305 (6)	C10—H10C	0.9600
O3—H3O	0.8400	C11—C12	1.522 (7)
O4—C4	1.210 (6)	C11—H11A	0.9700
O1W—H1W1	0.8400	C11—H11B	0.9700
O1W—H1W2	0.8400	C12—C13	1.541 (6)
O1W'—H1W3	0.8400	C12—H12	0.9800
O1W'—H1W4	0.8400	C13—H13A	0.9600
N1—C5	1.333 (6)	C13—H13B	0.9600
N1—C2	1.388 (5)	C13—H13C	0.9600
N2—C5	1.331 (6)	C14—C15	1.508 (6)
N2—C3	1.366 (6)	C14—H14A	0.9700
N3—C6	1.470 (6)	C14—H14B	0.9700
N3—C20	1.493 (5)	C15—H15A	0.9700
N3—H3N	0.8600	C15—H15B	0.9700
N4—C8	1.485 (6)	C16—C17	1.515 (6)
N4—C7	1.489 (5)	C16—C19	1.533 (7)
N4—H4N	0.8600	C16—C18	1.541 (7)
N5—C14	1.471 (6)	C17—H17A	0.9600
N5—C12	1.484 (5)	C17—H17B	0.9600
N5—H5N	0.8600	C17—H17C	0.9600
N6—C15	1.480 (6)	C18—H18A	0.9600
N6—C16	1.511 (6)	C18—H18B	0.9600
N6—H6N	0.8600	C18—H18C	0.9600
C1—C2	1.458 (7)	C19—C20	1.513 (7)
C2—C3	1.404 (6)	C19—H19A	0.9700
C3—C4	1.487 (7)	C19—H19B	0.9700
C5—H5	0.9300	C20—C21	1.535 (6)
C6—C7	1.511 (6)	C20—H20	0.9800
C6—H6A	0.9700	C21—H21A	0.9600
C6—H6B	0.9700	C21—H21B	0.9600
C7—H7A	0.9700	C21—H21C	0.9600
C7—H7B	0.9700		
			
N5—Ni1—N1	95.99 (14)	C10—C8—C11	107.7 (4)
N5—Ni1—N3	99.73 (13)	C8—C9—H9A	109.5
N1—Ni1—N3	162.72 (14)	C8—C9—H9B	109.5
N5—Ni1—O1	169.03 (13)	H9A—C9—H9B	109.5
N1—Ni1—O1	78.92 (13)	C8—C9—H9C	109.5
N3—Ni1—O1	86.74 (13)	H9A—C9—H9C	109.5
N5—Ni1—N4	87.46 (14)	H9B—C9—H9C	109.5
N1—Ni1—N4	103.71 (14)	C8—C10—H10A	109.5
N3—Ni1—N4	84.20 (14)	C8—C10—H10B	109.5
O1—Ni1—N4	84.36 (13)	H10A—C10—H10B	109.5
N5—Ni1—N6	84.52 (14)	C8—C10—H10C	109.5
N1—Ni1—N6	87.00 (14)	H10A—C10—H10C	109.5
N3—Ni1—N6	87.40 (14)	H10B—C10—H10C	109.5
O1—Ni1—N6	104.75 (13)	C12—C11—C8	119.7 (4)
N4—Ni1—N6	167.25 (14)	C12—C11—H11A	107.4
C1—O1—Ni1	115.5 (3)	C8—C11—H11A	107.4
C4—O3—H3O	109.5	C12—C11—H11B	107.4
H1W1—O1W—H1W2	111.3	C8—C11—H11B	107.4
H1W3—O1W'—H1W4	114.2	H11A—C11—H11B	106.9
C5—N1—C2	104.5 (4)	N5—C12—C11	111.1 (4)
C5—N1—Ni1	145.8 (3)	N5—C12—C13	112.7 (4)
C2—N1—Ni1	109.7 (3)	C11—C12—C13	109.4 (4)
C5—N2—C3	103.8 (4)	N5—C12—H12	107.8
C6—N3—C20	112.9 (4)	C11—C12—H12	107.8
C6—N3—Ni1	104.3 (2)	C13—C12—H12	107.8
C20—N3—Ni1	115.0 (3)	C12—C13—H13A	109.5
C6—N3—H3N	108.1	C12—C13—H13B	109.5
C20—N3—H3N	108.1	H13A—C13—H13B	109.5
Ni1—N3—H3N	108.1	C12—C13—H13C	109.5
C8—N4—C7	114.2 (3)	H13A—C13—H13C	109.5
C8—N4—Ni1	122.1 (3)	H13B—C13—H13C	109.5
C7—N4—Ni1	104.4 (3)	N5—C14—C15	110.3 (4)
C8—N4—H4N	104.9	N5—C14—H14A	109.6
C7—N4—H4N	104.9	C15—C14—H14A	109.6
Ni1—N4—H4N	104.9	N5—C14—H14B	109.6
C14—N5—C12	112.7 (3)	C15—C14—H14B	109.6
C14—N5—Ni1	105.4 (2)	H14A—C14—H14B	108.1
C12—N5—Ni1	116.3 (3)	N6—C15—C14	110.2 (4)
C14—N5—H5N	107.4	N6—C15—H15A	109.6
C12—N5—H5N	107.4	C14—C15—H15A	109.6
Ni1—N5—H5N	107.4	N6—C15—H15B	109.6
C15—N6—C16	114.7 (3)	C14—C15—H15B	109.6
C15—N6—Ni1	103.8 (3)	H15A—C15—H15B	108.1
C16—N6—Ni1	121.2 (3)	N6—C16—C17	107.4 (4)
C15—N6—H6N	105.2	N6—C16—C19	109.6 (4)
C16—N6—H6N	105.2	C17—C16—C19	111.8 (4)
Ni1—N6—H6N	105.2	N6—C16—C18	111.4 (4)
O1—C1—O2	124.0 (5)	C17—C16—C18	108.4 (4)
O1—C1—C2	116.5 (4)	C19—C16—C18	108.3 (4)
O2—C1—C2	119.5 (4)	C16—C17—H17A	109.5
N1—C2—C3	106.5 (4)	C16—C17—H17B	109.5
N1—C2—C1	119.2 (4)	H17A—C17—H17B	109.5
C3—C2—C1	134.3 (4)	C16—C17—H17C	109.5
N2—C3—C2	109.5 (4)	H17A—C17—H17C	109.5
N2—C3—C4	122.0 (4)	H17B—C17—H17C	109.5
C2—C3—C4	128.5 (5)	C16—C18—H18A	109.5
O4—C4—O3	121.0 (5)	C16—C18—H18B	109.5
O4—C4—C3	121.4 (5)	H18A—C18—H18B	109.5
O3—C4—C3	117.6 (5)	C16—C18—H18C	109.5
N2—C5—N1	115.6 (4)	H18A—C18—H18C	109.5
N2—C5—H5	122.2	H18B—C18—H18C	109.5
N1—C5—H5	122.2	C20—C19—C16	118.5 (4)
N3—C6—C7	110.0 (4)	C20—C19—H19A	107.7
N3—C6—H6A	109.7	C16—C19—H19A	107.7
C7—C6—H6A	109.7	C20—C19—H19B	107.7
N3—C6—H6B	109.7	C16—C19—H19B	107.7
C7—C6—H6B	109.7	H19A—C19—H19B	107.1
H6A—C6—H6B	108.2	N3—C20—C19	110.9 (4)
N4—C7—C6	108.7 (3)	N3—C20—C21	111.9 (4)
N4—C7—H7A	109.9	C19—C20—C21	110.4 (4)
C6—C7—H7A	109.9	N3—C20—H20	107.9
N4—C7—H7B	109.9	C19—C20—H20	107.9
C6—C7—H7B	109.9	C21—C20—H20	107.9
H7A—C7—H7B	108.3	C20—C21—H21A	109.5
N4—C8—C9	107.8 (4)	C20—C21—H21B	109.5
N4—C8—C10	113.0 (4)	H21A—C21—H21B	109.5
C9—C8—C10	107.4 (4)	C20—C21—H21C	109.5
N4—C8—C11	109.3 (4)	H21A—C21—H21C	109.5
C9—C8—C11	111.6 (4)	H21B—C21—H21C	109.5
			
N5—Ni1—O1—C1	−58.3 (8)	C5—N1—C2—C1	179.8 (4)
N1—Ni1—O1—C1	4.9 (3)	Ni1—N1—C2—C1	0.1 (5)
N3—Ni1—O1—C1	175.2 (3)	O1—C1—C2—N1	4.2 (6)
N4—Ni1—O1—C1	−100.3 (3)	O2—C1—C2—N1	−176.7 (4)
N6—Ni1—O1—C1	88.8 (3)	O1—C1—C2—C3	−177.8 (5)
N5—Ni1—N1—C5	−11.8 (6)	O2—C1—C2—C3	1.2 (8)
N3—Ni1—N1—C5	143.7 (6)	C5—N2—C3—C2	0.7 (5)
O1—Ni1—N1—C5	178.1 (6)	C5—N2—C3—C4	178.6 (5)
N4—Ni1—N1—C5	−100.6 (6)	N1—C2—C3—N2	−1.3 (5)
N6—Ni1—N1—C5	72.4 (6)	C1—C2—C3—N2	−179.4 (5)
N5—Ni1—N1—C2	167.8 (3)	N1—C2—C3—C4	−179.1 (5)
N3—Ni1—N1—C2	−36.7 (6)	C1—C2—C3—C4	2.8 (9)
O1—Ni1—N1—C2	−2.3 (3)	N2—C3—C4—O4	1.5 (8)
N4—Ni1—N1—C2	79.0 (3)	C2—C3—C4—O4	179.0 (5)
N6—Ni1—N1—C2	−108.0 (3)	N2—C3—C4—O3	−178.7 (5)
N5—Ni1—N3—C6	−103.8 (3)	C2—C3—C4—O3	−1.2 (8)
N1—Ni1—N3—C6	101.1 (5)	C3—N2—C5—N1	0.3 (6)
O1—Ni1—N3—C6	67.3 (3)	C2—N1—C5—N2	−1.1 (5)
N4—Ni1—N3—C6	−17.3 (3)	Ni1—N1—C5—N2	178.5 (4)
N6—Ni1—N3—C6	172.3 (3)	C20—N3—C6—C7	171.3 (3)
N5—Ni1—N3—C20	132.0 (3)	Ni1—N3—C6—C7	45.7 (4)
N1—Ni1—N3—C20	−23.2 (6)	C8—N4—C7—C6	176.8 (4)
O1—Ni1—N3—C20	−56.9 (3)	Ni1—N4—C7—C6	41.0 (4)
N4—Ni1—N3—C20	−141.6 (3)	N3—C6—C7—N4	−61.4 (5)
N6—Ni1—N3—C20	48.0 (3)	C7—N4—C8—C9	163.5 (4)
N5—Ni1—N4—C8	−44.3 (3)	Ni1—N4—C8—C9	−69.3 (4)
N1—Ni1—N4—C8	51.3 (3)	C7—N4—C8—C10	45.0 (5)
N3—Ni1—N4—C8	−144.3 (3)	Ni1—N4—C8—C10	172.2 (3)
O1—Ni1—N4—C8	128.4 (3)	C7—N4—C8—C11	−75.0 (5)
N6—Ni1—N4—C8	−95.3 (7)	Ni1—N4—C8—C11	52.2 (4)
N5—Ni1—N4—C7	87.1 (3)	N4—C8—C11—C12	−60.0 (5)
N1—Ni1—N4—C7	−177.4 (2)	C9—C8—C11—C12	59.2 (6)
N3—Ni1—N4—C7	−13.0 (3)	C10—C8—C11—C12	176.8 (4)
O1—Ni1—N4—C7	−100.3 (3)	C14—N5—C12—C11	173.0 (3)
N6—Ni1—N4—C7	36.1 (7)	Ni1—N5—C12—C11	−65.2 (4)
N1—Ni1—N5—C14	70.1 (3)	C14—N5—C12—C13	49.8 (5)
N3—Ni1—N5—C14	−102.7 (3)	Ni1—N5—C12—C13	171.6 (3)
O1—Ni1—N5—C14	131.8 (6)	C8—C11—C12—N5	68.9 (5)
N4—Ni1—N5—C14	173.6 (3)	C8—C11—C12—C13	−166.0 (4)
N6—Ni1—N5—C14	−16.3 (3)	C12—N5—C14—C15	171.1 (3)
N1—Ni1—N5—C12	−55.5 (3)	Ni1—N5—C14—C15	43.4 (4)
N3—Ni1—N5—C12	131.7 (3)	C16—N6—C15—C14	174.2 (4)
O1—Ni1—N5—C12	6.2 (9)	Ni1—N6—C15—C14	39.7 (4)
N4—Ni1—N5—C12	48.0 (3)	N5—C14—C15—N6	−58.8 (5)
N6—Ni1—N5—C12	−141.9 (3)	C15—N6—C16—C17	164.8 (4)
N5—Ni1—N6—C15	−12.6 (3)	Ni1—N6—C16—C17	−69.3 (4)
N1—Ni1—N6—C15	−109.0 (3)	C15—N6—C16—C19	−73.6 (5)
N3—Ni1—N6—C15	87.4 (3)	Ni1—N6—C16—C19	52.4 (5)
O1—Ni1—N6—C15	173.3 (3)	C15—N6—C16—C18	46.2 (5)
N4—Ni1—N6—C15	38.6 (7)	Ni1—N6—C16—C18	172.2 (3)
N5—Ni1—N6—C16	−143.4 (3)	N6—C16—C19—C20	−62.3 (5)
N1—Ni1—N6—C16	120.3 (3)	C17—C16—C19—C20	56.6 (5)
N3—Ni1—N6—C16	−43.3 (3)	C18—C16—C19—C20	176.0 (4)
O1—Ni1—N6—C16	42.6 (3)	C6—N3—C20—C19	173.2 (4)
N4—Ni1—N6—C16	−92.1 (7)	Ni1—N3—C20—C19	−67.2 (4)
Ni1—O1—C1—O2	174.8 (3)	C6—N3—C20—C21	49.6 (5)
Ni1—O1—C1—C2	−6.2 (5)	Ni1—N3—C20—C21	169.1 (3)
C5—N1—C2—C3	1.4 (5)	C16—C19—C20—N3	72.5 (5)
Ni1—N1—C2—C3	−178.4 (3)	C16—C19—C20—C21	−163.0 (4)

### Hydrogen-bond geometry (Å, °)

**Table:**

*D*—H···*A*	*D*—H	H···*A*	*D*···*A*	*D*—H···*A*
O3—H3o···O2	0.84	1.64	2.468 (5)	170
N5—H5n···O4^i^	0.86	2.14	2.973 (5)	162

Symmetry codes: (i) *x*−1, *y*, *z*.
